# First Draft Genome for Red Sea Bream of Family Sparidae

**DOI:** 10.3389/fgene.2018.00643

**Published:** 2018-12-12

**Authors:** Ga-Hee Shin, Younhee Shin, Myunghee Jung, Ji-man Hong, Sangmin Lee, Sathiyamoorthy Subramaniyam, Eun-Soo Noh, Eun-Ha Shin, Eun-Hee Park, Jung Youn Park, Young-Ok Kim, Kwnag-Min Choi, Bo-Hye Nam, Chan-Il Park

**Affiliations:** ^1^Research and Development Center, Insilicogen Inc., Yongin-si, South Korea; ^2^Department of Biological Sciences, Sungkyunkwan University, Suwon, South Korea; ^3^Biotechnology Research Division, National Institute of Fisheries Science, Busan, South Korea; ^4^Department of Marine Biology and Aquaculture, College of Marine Science, Gyeongsang National University, Tongyeong, South Korea

**Keywords:** *Pagrus major*, genome, PacBio, Sparidae, red sea bream

## Introduction

Reference genomes for all organisms on earth are now attainable owing to advances in genome sequencing technologies (Goodwin et al., 2016). Generally, species that contribute considerably to the economy or human welfare are sequenced and are considered more important than others. Furthermore, coastal indigenous people mainly depend on marine species for their food sources, which has resulted in the extinction of several marine species (Cisneros-Montemayor et al., 2016). Of these, an extinction risk assessment of marine fishes, mainly for sea breams (Family: Sparidae), has recently been conducted by way of a global extinction risk assessment from the dataset of the International Union for Conservation of Nature's Red List Process, which mentions that around 25 species are threatened/near-threatened according to their body weight (Comeros-Raynal et al., 2016). Another report clearly showed the benefit of worldwide aquaculture production, which contributed to 47% of total seafood production, and also highlighted the over-fishing of sea breams (FAO, 2018). The Republic of Korea is the fourth largest seafood producer in the world, producing 3.3 million tons in 2015 and exporting seafood worth $1.6 billion in 2016; therefore, aquaculture-associated research is fundamental for Korea. In the present study, the red sea bream (*Pagrus major*), which belongs to the family Sparidae, which comprises 35 genera, 132 species, and 10 subspecies (de la Herran et al., 2001; NCBI, 2018), was assessed. It is widely distributed in the coastal regions of Korea, Japan, China, and Taiwan (Blanco Gonzalez et al., 2015), commonly on rocky substrates, soft sand, and muddy bottoms. Species of this family are hermaphroditic and mature 4 years after birth, surviving for 10 or more years. This group of fishes is an important resource to better understand the genetics of sexual dimorphism. Another major factor affecting this species is microbial infections, which are dominant in the aquaculture industry and account for a considerable decline in aquaculture production (Nam et al., 2016; Sawayama et al., 2017). Few studies have analyzed the molecular markers associated with these problems. Recently, sexual dimorphism-related genes from the *Sparus aurata* genome have been profiled, including stage-specific expression (Pauletto et al., 2018), and three other studies have assessed molecular markers associated with microbial and environmental toxicity in the red sea bream (Iida et al., 2016; Hano et al., 2017; Sawayama et al., 2017). However, genome-wide molecular marker characterization is needed to conduct genome selection in breeding schemes (López et al., 2014), which is not possible in *P. major*, owing to the absence of a reference genome. To the best of our knowledge, only two draft genomes (*S. aurata* and *Spondyliosoma cantharus*) are available for the entire Sparidae family, which is the largest clade in class Actinopteri (de la Herran et al., 2001), but there is no draft or reference genome sequence for the genus *Pagrus*. Therefore, we constructed a draft genome using contig level assembly, with a size of 829.3 Mb, employing the 90X PacBio sequence alone.

### Value of the Data

This draft genome would be considerably useful for detailing the molecular characterization of various breeding-associated problems in species from the family Sparidae as well as other comparative genome mining applications.

## Materials and Methods

### Sample Collection and Genomic DNA Extraction

A single female fish (4.25 kg) was collected on December 2016 from the Jeju Fisheries Research Institute and maintained at 22 ± 0.5°C in aerated seawater (NFRDI-2016-01-2). The abdominal muscle tissues were sampled aseptically and stored in liquid nitrogen for genomic DNA extraction. The complete experimental procedure, from DNA isolation to sequencing, was conducted using DNALink, South Korea (www.dnalink.com), as instructed in the respective product protocols.

### Genomic DNA Library Preparation and Sequencing

Highly concentrated genomic DNA (gDNA) (24 μg) from each given sample was prepared using a DNeasy Animal Mini Kit (Qiagen, Hilden, Germany). The complete isolated gDNA was quantified using a ND-1000 spectrophotometer (Thermo Scientific, Wilmington, DE, USA) and Qubit fluorometer. The total gDNA were subjected to other steps i.e., fragmentation with Covaris G-Tube to obtain > 20 KB fragments, filtering of small fragments using 0.45X AMPure®, fragment end repair using ExoVII, ligation of blunt adapters using double standard DNA fragments, attachment of the primer and polymerase to the SMRTbell^TM^ templates (Template Prep Kit 1.0), and the addition of MagBeads. Finally, the impurities were washed out carefully with 1.0X AMPure® and only the double stranded DNA fragments with blunt adapters were subjected to sequencing using C4-chemistry (DNA sequencing Reagent 4.0) in the PacBio (Pacific Biosciences) sequencing platform by capturing a movie for 1 × 240 min of each SMRT cell. Similarly, the isolated gDNAs were also subjected to sequencing library preparation with stranded Illumina paired-end (PE) protocols (Illumina, San Diego, CA, USA). The fragmented libraries were subjected to size selection and sequenced with an Illumina Hiseq 2000 sequencer.

### Illumina Pre-process and Genome Size Estimation

Full Illumina DNA sequences were subjected to pre-processing steps, which included adapter trimming, quality trimming (Phred(Q) ≥ 20), and contamination removal. The adapter and quality trims were conducted using Trimmomatic-0.32 functions (Bolger et al., 2014), and the microbial contamination of each sample was removed using CLCMapper v4.2.0 (www.qiagenbioinformatics.com) with an in-house database. Here, the in-house database was constructed from bacterial (ftp://ftp.ncbi.nlm.nih.gov/genomes/GENOME_REPORTS/prokaryotes.txt), viral (ftp://ftp.ncbi.nlm.nih.gov/genomes/Viruses/), and marine (https://www.ncbi.nlm.nih.gov/bioproject/PRJNA13694) metagenomes. All the pre-processed sequences from the paired-end library were subjected to genome size estimation using the *k*-mer based method (which was used in the panda genome Li et al., 2009. The *k*-mer frequencies (*k*-mer size = 19) were obtained using the Jellyfish v2.0 method (Marçais and Kingsford, 2011), and the genome size was calculated from the given formulas: Genome Coverage Depth = (*k*-mer Coverage Depth X Average Read Length) / (Average Read Length – *k*-mer size +1) and Genome size = Total Base Number / Genome Coverage Depth. Alternatively, the PacBio sequences were only subjected to error correction using CLCAssemblyCell v4.2.0.

### PacBio Error-Correction and *de-novo* Genome Assembly

Complete PacBio sequence reads were processed for error correction (Read Quality ≥ 0.75 and Read Length ≥ 50) with processed Illumina short reads using SMRTAnalysis v2.3 and the error corrected PacBio reads were imported to a diploid-aware hierarchical genome assembler to construct the contigs from the long-sequence PacBio reads, i.e., FALCON (Chin et al., 2016). The assembled contigs were further subjected to sequence polishing using the Quiver consensus method to reduce the base called errors (Chin et al., 2013). Finally, the assembled and polished contigs were assessed to determine genome completeness using BUSCO v3.0 (Simão et al., 2015). The reference BUSCO datasets used were vertebrata_odb9 and actinopterygii_odb9. The quality of the assembly was assessed by short-reads mapping to the draft using CLCMapper v5.0.4.

### *De novo* Repeat Region Prediction and Classification

The repeat regions were predicted using the *de novo* method and classified into repeat subclasses. The *de novo* repeat prediction for *P. major* was conducted using RepeatModeler (www.repeatmasker.org/RepeatModeler/), which includes other methods such as RECON (Bao and Eddy, 2002) (http://eddylab.org/software/recon/), RepeatScout (Price et al., 2005) (https://bix.ucsd.edu/repeatscout/), and TRF (Benson, 1999) (https://tandem.bu.edu/trf/trf.html). The modeled repeats were classified into their subclasses using the reference Repbase v20.08 database (www.girinst.org/repbase/) (Bao et al., 2015) and these repeats were masked using RepeatMasker v4.0.5 (www.repeatmasker.org) with RMBlastn v2.2.27^+^.

### Gene Prediction and Annotation

The genes from the *P. major* draft were predicted using an in-house gene prediction pipeline, which includes three modules: an evidence-based gene modeler (EVM), an *ab-initio* gene modeler, and a consensus gene modeler. Finally, functional annotation processing was conducted for the consensus genes. Initially, sequenced transcriptomes from two methods [Illumina (186.6 Gb) and IsoSeq (1.2 Gb)] were mapped to the *P. major* repeat masked draft genome using Tophat (Trapnell et al., 2012) and the transcripts/gene structural boundaries were predicted using Cufflink (Trapnell et al., 2012) and PASA (Haas et al., 2003). To train the *ab-initio*, gene modeler and EVM (which includes Exonerate Slater and Birney, 2005, AUGUSTUS Stanke et al., 2006 and GENEID Blanco et al., 2007), as well as several genomes (*Danio rerio, Gasterosteus aculeatus, Tetraodon nigroviridis, Takifugu rubripes, Oryzias latipes, Notothenia coriiceps, Haplochromis burtoni, Stegastes partitus, Sebastes schlegelii, Oplegnathus fasciatus*, and *Homo sapiens*) were used for prediction. Finally, the predicted gene and transcript models from the EVM and *ab-initio* modeler were subjected to the consensus gene modeler (which includes EVidenceModeler, Haas et al., 2008) to produce the final gene and transcript models. Finally, the consensus transcripts were subjected to functional annotation from biological databases (NCBI-NR databases, Swiss-Prot, Gene Ontologies and KEGG pathways) using Blast2GO (Götz et al., 2008).

### Preliminary Analysis Report

**Figure 1:**
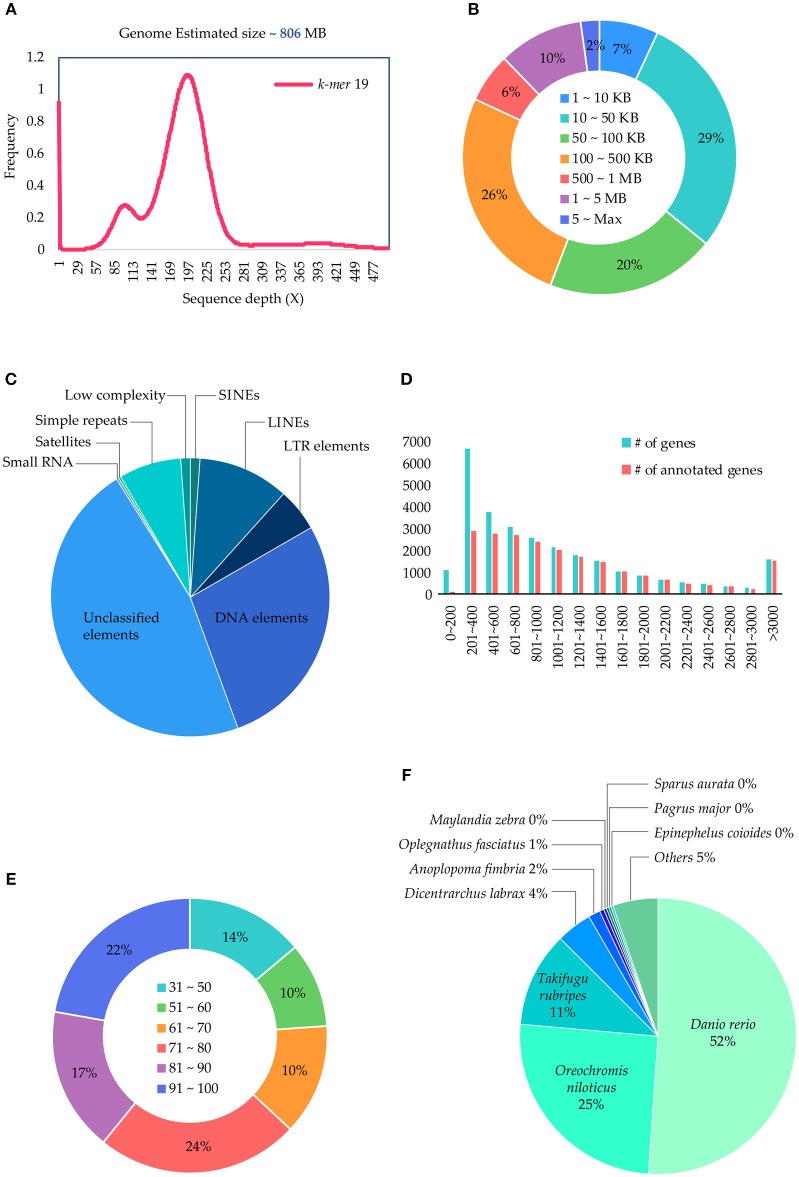
Illustration of genome size, genes, and functional elements of the *Pagrus major* genome. **(A)** *k-*mer based genome size estimation; **(B)** assembled contigs length distributions; **(C)** *de-novo* repeat predictions and sub-class distributions; **(D)** length distribution of the predicted genes with their functional annotation status; **(E)** sequence blast similarity score distribution, and **(F)** species distribution obtained from BLAST.

The *P. major* genome size was estimated as ~806 Mb (Figure 1A) using the *k-*mer method from 190.3 Gb of the short-read sequences (Table 1), which were generated using the Illumina sequencer. The 73 Gb long-read sequences, which were generated using the PacBio sequencer, were assembled into 1,657 contigs with a total size of 829.3 Mb and an N50 of 2.8 Mb (Table 1), and 92.6% of the paired short-reads were mapped correctly to the assembled contigs, which clearly showed the assembly quality. Particularly, 12% of the contigs were > 1Mb in length (Figure 1B) and < 7% of the contigs were < 10 Kb in size (Figure 1B). The repeat contents in the genome were 257 Mb (31.1%) bases, which were predicted and classified into their sub-classes (Figure 1C). In this genome, 28,343 consensus genes were predicted with an average length of 5,913 bp (Table 1, section C) and, among those, 76.2% of the genes obtained annotations from the Uniprot database (Figure 1E). Most of the short genes were left unannotated compared to others (Figure 1D). Moreover, 52% of the annotated genes obtained annotation from the fish *Danio rerio* (Figure 1F). Additionally, BUSCO scores were obtained for the two datasets: 97.8% (2,529/2,586) in vertebrata and 97.1% in actinopterygii (4,447/4,584), which shows the confidence of the completeness of the annotated genes in the assembled genome. Therefore, we propose that this draft version is a near-complete reference genome for *P. major* and, in comparison with 68 other available genome assemblies for the bony fish clade (Percomorphaceae) in the NCBI assembly (lastly accessed: March 2018), this draft is assembled well at the contig level. Moreover, this is the best assembled draft for the genus *Pagrus* and family Sparidae at the contig level and will be good as a base to improve scaffold/chromosomal-level genome assemblies and as a reference for other functional studies.

**Table 1:** Summary of genome assemblies and gene annotations.

**Technology**	**Illumina**	**PacBio**
**A. SEQUENCES**		
Raw data in Gb (Coverage)	190.3 (~240 X)	73.0 (~90 X)
Pre-processed data in Gb (%)	156.4 (82%)	73.0 (100%)
**B. ASSEMBLY**		
No of Contigs	1,657	
Total Bases	829,318,935	
Average length	500,494	
Minimum length	153	
Maximum length	12,966,191	
N50	2,896,215	
N (%)	0	
GC (%)	41.23	
**C. GENE**		
# of genes	28,343 (6.24 exons/ gene)	
Average gene length	5,913 bp	
Average exon length	178 bp	
Repeat elements	31.11%	
Genome coverage (gene region)	20.20%	
**D. ANNOTATIONS**		
Blast hits	21,605 (76.22%)	
No hits	6,738 (23.77%)	

### Deposited Data and Information to the User

The complete sequences, which were used for the genome assemblies and annotations, have been deposited in public data repositories. The DNA libraries used in the current draft genome assembly for *P. major* have been deposited in the NCBI sequence read archive (Project ID: PRJNA480768) and the structural and functional annotation (CDS, gff, repeat regions, and proteins) datasets have been deposited in the figshare repository (doi: 10.6084/m9.figshare.6962867.v1). The format and description of all the deposited datasets are mentioned in the readme file, which have been deposited in the figshare repository.

## Author Contributions

G-HS, YS, MJ, JH, SL, and SS: genome assembly and annotations. SS and YS: manuscript preparation. E-SN, E-HS, E-HP, JP, Y-OK, and K-MC: sampling and sequencing. B-HN and C-IP: funding and modeled the study.

### Conflict of Interest Statement

The authors declare that the research was conducted in the absence of any commercial or financial relationships that could be construed as a potential conflict of interest.
